# Reduction of Pulmonary Hypertension After Transition to Sacubitril/Valsartan in Patients With Heart Failure With Preserved Ejection Fraction

**DOI:** 10.3389/fcvm.2021.734697

**Published:** 2021-10-07

**Authors:** Christof Burgdorf, Janine Brockmöller, Henrieke Strampe, Monika Januszewski, Bjoern Andrew Remppis

**Affiliations:** Department of Cardiology, Heart and Vascular Center Bad Bevensen, Bad Bevensen, Germany

**Keywords:** Sacubitril/Valsartan, HFpEF, pulmonary hypertension, right heart catheterisation, case series

## Abstract

**Objectives:** Although the PARAGON-HF trial failed to reach its primary endpoint, subgroups of patients with heart failure with preserved ejection fraction (HFpEF) still appear to benefit from Sacubitril/Valsartan therapy. As HFpEF patients with pulmonary hypertension display a specifically high mortality and morbidity, we evaluated the effect of Sacubitril/Valsartan in this subgroup of HFpEF patients.

**Methods:** In this retrospective case-series of 18 patients with HFpEF and pulmonary hypertension, right heart catheterisation (RHC) for determination of invasive pulmonary pressure were performed at baseline (pre-Sacubitril/Valsartan) and 99 (71–156) days after transition from angiotensin-converting enzyme inhibitors and angiotensin receptor blockers to Sacubitril/Valsartan (post-Sacubitril/Valsartan). Results are given as median and interquartile range.

**Results:** After conversion to Sacubitril/Valsartan, RHC showed significantly reduced pulmonary artery pressure (PAP) and mean pulmonary capillary wedge pressure (PCWP) compared to pre-Sacubitril/Valsartan [PAP systolic/diastolic/mean 44 (38–55)/15 (11–20)/27 (23–33) mm Hg vs. 51 (41–82)/22 (13–29)/33 (28–52) mm Hg, *p* < 0.05 and *p* < 0.01, respectively; PCWP 16 (12–20) mm Hg vs. 22 (15–27) mm Hg, *p* < 0.05]. Median Sacubitril/Valsartan dosage was 24/26 mg BID (24/26 BID−49/51 mg BID). Clinically, New York Heart Association functional class improved in 12 of the 18 patients (*p* < 0.01) after conversion to Sacubitril/Valsartan. Echocardiographic parameters of left ventricular function and cardiovascular co-medication did not differ markedly between pre- and post-Sacubitril/Valsartan.

**Conclusion:** Sacubitril/Valsartan therapy is associated with an improvement of pulmonary hypertension in HFpEF patients.

## Introduction

Heart failure with preserved ejection fraction (HFpEF) is by now the most common form of heart failure and its incidence still continues to grow worldwide, but an approved guideline therapy is still not available (1). Only recently, the PARAGON-HF trial failed to unveil a significant benefit of angiotensin receptor-neprilysin inhibition with Sacubitril/Valsartan in HFpEF patients with respect to its primary composite outcome of total hospitalization for heart failure and death from cardiovascular causes (2). Notwithstanding, this trial offers important implications as pre-specified subgroups of patients were identified that still might benefit from Sacubitril/Valsartan therapy. Females and patients with a moderately decreased ejection fraction or kidney function showed a significantly improved primary outcome as outlined by Solomon et al. (3).

In rat models of pulmonary hypertension, Sacubitril/Valsartan has been shown to reduce pulmonary pressures and histological vascular remodeling (4, 5) and others have shown that Sacubitril/Valsartan lowers pulmonary pressure in patients with heart failure with reduced ejection fraction (HFrEF) (6, 7). HFpEF patients with pulmonary hypertension as a comorbid condition carry a significantly elevated long-term mortality risk and suffer the most as far as dyspnea is concerned (8).

We therefore sought to investigate whether Sacubitril/Valsartan would impact pulmonary hypertension in this subgroup of otherwise heterogeneous HFpEF patients diagnosed according to the HFA-PEFF algorithm (9).

## Methods

This is a retrospective case-series of 18 patients with HFpEF and dyspnoea as the leading symptom for hospital admission (January 2018 to August 2020) in whom Sacubitril/Valsartan was initiated at the discretion of the treating physician. Determination of invasive pulmonary pressure was performed by right heart catheterisation (RHC) at baseline in the absence of Sacubitril/Valsartan (pre-Sacubitril/Valsartan). Following this, angiotensin-converting enzyme inhibitors and angiotensin receptor blockers were converted to Sacubitril/Valsartan at 24/26 mg BID and was titrated to the highest clinically and hemodynamically tolerated dose. Effects of Sacubitril/Valsartan on pulmonary pressures were documented 99 (71–156) days later during a planned second hospital admission with follow-up RHC (post-Sacubitril/Valsartan).

All RHC were performed in the catheterisation laboratory with a Philips Allura Xper FD10 cardiovascular X-ray system including Xper Flex Cardio Physiomonitoring System for recording of end-expiratory pulmonary artery pressure (PAP), mean pulmonary capillary wedge pressure (PCWP), right atrial (RA) pressure and calculation of pulmonary vascular resistance (PVR), cardiac output (CO), cardiac index (CI), and systemic vascular resistance (SVR). CO was calculated according to Fick's principle. Pre- and post-Sacubitril/Valsartan RHC were performed by the same cardiologist each and transfemoral venous access using a 7F Swan-Ganz catheter was the preferred approach. HFpEF was diagnosed according to the recommendations from the Heart Failure Association of the European Society of Cardiology (9).

Statistical analysis was performed using GraphPad Prism 7.0 (GraphPad Software Inc., San Diego, CA, USA). Continuous data are presented as median and interquartile range; frequencies and percentages are used to describe discrete variables. Continuous data were analyzed using the Wilcoxon matched-pairs signed rank test and categorical variables using the two-tailed Fisher's exact-test. A *p*-value of < 0.05 was considered statistically significant.

## Results

**Figure 1:**
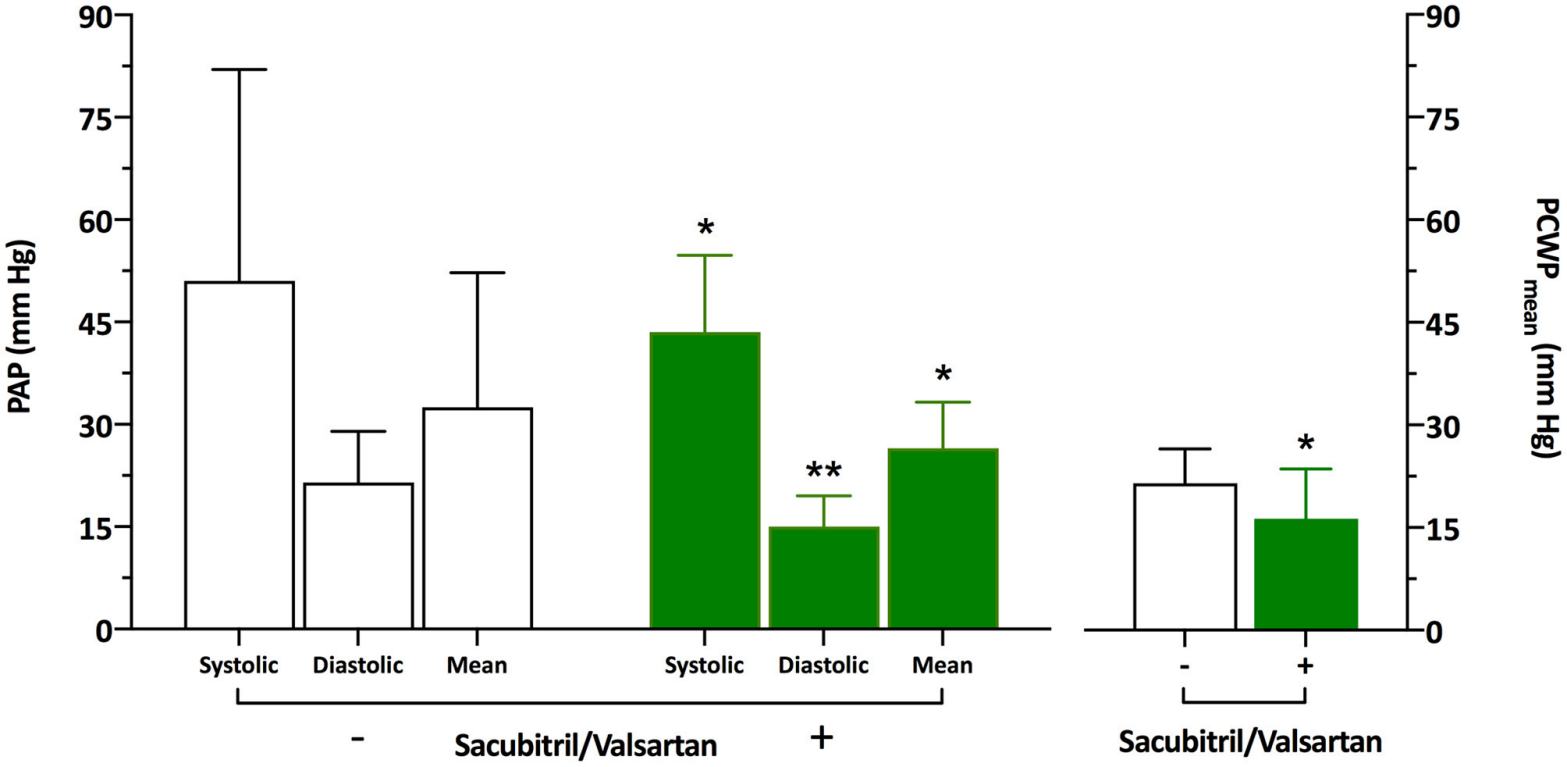
Pulmonary artery pressure (PAP) and mean pulmonary capillary wedge pressure (PCWP) pre (–) and post (+) medication with Sacubitril/Valsartan [24/26 mg BID (24/26 BID−49/51 BID)] (*n* = 18). Data are given as median and interquartile range. *p < 0.05, **p < 0.01.

Clinical patient characteristics are summarized in Table 1. Thirteen of the 18 patients presented with New York Heart Association (NYHA) functional class III, the remaining five patients presented with NYHA functional class IV. Total HFA-PEFF of the entire cohort was 6 (5–6). Pre-Sacubitril/Valsartan RHC [performed on hospital day 1 (0–3)] revealed elevated pulmonary artery pressure (PAP) [systolic/diastolic/mean 51 (41–82)/22 (13–29)/33 (28–52) mm Hg], mean pulmonary capillary wedge pressure (PCWP) [22 (15–27) mm Hg], and pulmonary vascular resistance (PVR) [200 (112–398) dyn·s/cm^5^] (Figure 1). After conversion to Sacubitril/Valsartan, follow-up RHC [performed on hospital day 1 (0–2) after readmission] showed significantly reduced PAP [systolic/diastolic/mean 44 (38–55)/15 (11–20)/27 (23–33) mm Hg] and mean PCWP [16 (12–20) mm Hg] compared to pre-Sacubitril/Valsartan (Figure 1). Median Sacubitril/Valsartan dosage at follow-up was 24/26 mg BID (24/26 BID−49/51 mg BID). Clinically, NYHA functional class had improved by at least one class in 12 of the 18 patients (*p* < 0.01). Invasive determination further revealed a markedly reduced RA pressure at follow-up [8 (5–9) mm Hg vs. 11 (4–19) mm Hg pre-Sacubitril/Valsartan, *p* < 0.05] presumably as a consequence of decreased PAP. Other invasive parameters such as PVR, CO, CI, and SVR did not differ significantly between pre- and post-Sacubitril/Valsartan, respectively [PVR: 200 (112–398) dyn·s/cm^5^ vs. 161 (101–221) dyn·s/cm^5^, CO: 5.0 (3.9–5.8) l/min vs. 4.7 (3.6–6.2) l/min, CI: 2.4 (2.1–3.1) l/min/m^2^ vs. 2.5 (1.8–3.1) l/min/m^2^, SVR: 1,528 (1,140–1,948) dyn·s/cm^5^ vs. 1,588 (1,131–1,824) dyn·s/cm^5^]. Furthermore, echocardiographic parameters of left ventricular function and cardiovascular co-medication did not differ significantly between pre- and post-Sacubitril/Valsartan (Table 1).

**Table 1:** Patient characteristics (*n* = 18).

	**Pre-Sac/Val**	**Post-Sac/Val**
Age (years)	73 (68–71)	
Female: male (*n*)	9:9	
Body weight (kg)	91.5 (74.4–112.8)	90.7 (73.3–106.9)
Mean arterial pressure (mm Hg)	99 (90–105)	90 (87–101)
Heart rate (bpm)	65 (61–72)	64 (59–69)
**Cardiovascular risk factors**		
Arterial hypertension (*n*)	18 (100%)	
Diabetes mellitus (*n*)	7 (38.9%)	
Hyperlipidaemia (*n*)	11 (61.1%)	
Smoking (*n*)	6 (33.3%)	
Family history for CAD (*n*)	3 (16.7%)	
Body mass index (kg/m^2^)	32.4 (28.4–37.5)	
CAD (*n*)	9 (50.0%)	
Prior MI (*n*)	-	
Prior PCI (*n*)	1 (5.6%)	
Prior CABG (*n*)	2 (11.1%)	
**ECG**		
Sinus rhythm (*n*)	12 (66.7%)	
Atrial fibrillation (*n*)	4 (22.2%)	
Pacemaker (*n*)	4 (22.2%)	
**TTE/TOE**		
LVEF (%)	64 (60–67)	60 (59–68)
GLS (%)	−18 (−20 to −15)	−20 (−21 to −18)
LVEDV (ml)	78 (65–106)	80 (65–121)
TAPSE (cm)	1.8 (1.5–2.3)	1.9 (1.6–2.3)
RV diameter (cm)	4.1 (3.3–4.3)	4.0 (3.6–4.1)
Mitral regurgitation ≥ II (*n*)	3 (16.7%)	1 (5.6%)
Tricuspid regurgitation ≥ II (*n*)	6 (33.3%)	3 (16.7%)
NT-proBNP (ng/l)	1,143 (574–1,856)	680 (269–1,523)
Creatinine (mg/dl)	1.0 (0.8–1.4)	1.0 (0.8–1.3)
GFR (ml/min)	60 (49–60)	58 (45–60)
**Medical treatment**		
Sacubitril/Valsartan (*n*)	-	18 (100%)
ACE inhibitors (*n*)	5 (27.8%)	-
Ramipril (mg/day)	10 (5–10)	-
ARBs (*n*)	13 (72.2 %)	-
Valsartan (mg/day)	160 (80–240)	-
Candesartan (mg/day)	32 (16–32)	-
Beta blockers (*n*)	15 (83.3%)	15 (83.3%)
Metoprolol (mg/day)	190 (95–190)	190 (100–190)
Bisoprolol (mg/day)	2.5 (2.5–5.0)	5 (2.5–7.5)
Nebivolol (mg/day)	-	10 (10–10)
Carvedilol (mg/day)	-	50 (50–50)
Loop diuretics (*n*)	15 (83.3%)	15 (83.3%)
Furosemide (mg/day)	80 (80–80)	-
Torasemide (mg/day)	10 (7.5–20)	10 (10–20)
Thiazide diuretics (*n*)	1 (5.6%)	1 (5.6%)
Hydrochlorothiazide (mg/day)	25 (25–25)	-
Xipamide (mg/day)	-	10 (10–10)
Aldosterone antagonists (*n*)	5 (27.8%)	9 (50.0%)
Spironolactone (mg/day)	25 (25–25)	25 (25–25)
Eplerenone (mg/day)	25 (25–25)	25 (12.5–25)

*Data are given as median and interquartile range. ACE, angiotensin-converting enzyme; ARBs, angiotensin receptor blockers; CABG, coronary artery bypass grafting; CAD, coronary artery disease; ECG, electrocardiogram; GFR, glomerular filtration rate; GLS, global longitudinal strain; LVEDV, left ventricular end-diastolic volume; LVEF, left ventricular ejection fraction; MI, myocardial infarction; NT-proBNP, N-terminal pro-brain natriuretic peptide; PCI, percutaneous coronary intervention; Pre-Sac/Val, pre-Sacubitril/Valsartan; Post-Sac/Val, post-Sacubitril/Valsartan; RV, right ventricular; TAPSE, tricuspid annular plane systolic excursion; TOE, transoesophageal echocardiogram; TTE, transthoracic echocardiogram*.

## Discussion

To the best of our knowledge, this is the first human study to demonstrate that Sacubitril/Valsartan therapy is associated with improvement of pulmonary hypertension in HFpEF patients by lowering pulmonary artery pressures. Interestingly, although patients only received the lowest approved dosage of Sacubitril/Valsartan when hemodynamic control measurements were performed, patients experienced a significant improvement not only concerning the hemodynamic readout but also concerning NYHA classification. Invasive hemodynamic data are scarce as to Sacubitril/Valsartan therapy because invasive phenotyping of patients was never included in randomized controlled trials. Cacciatore et al. recently reported a similar impact of Sacubitril/Valsartan on pulmonary hypertension in end stage HFrEF patients that were listed for heart transplantation (6). Thus, Sacubitril/Valsartan would improve pulmonary hypertension in a very heterogeneous population of heart failure patients irrespective of whether HFrEF or HFpEF patients are concerned. This raises the question as how Sacubitril/Valsartan impacts pulmonary hemodynamics on the molecular level. Because neprilysin is a ubiquitous endopeptidase with > 50 putative peptide substrates, the inhibition of neprilysin would thus lead to pleiotropic effects depending on the net effect on various peptides (10). Importantly, the inhibition of neprilysin not only leads to increased levels of biologically active brain natriuretic peptide that reinstalls the production of cGMP and its effects on hemodynamics and kidney function. It also inhibits the activation of endothelin and increases serum levels of biologically active apelin at the same time (11, 12). While endothelin is known to amplify pulmonary vasoconstriction, apelin increases endothelial dependent vasodilation and inhibits pulmonary artery muscle proliferation (13). Moreover, apelin is known to exert strong positive inotropic effects while inhibiting angiotensin II dependent signal transduction. Taken together, positive inotropic effects in left ventricular myocardium and vasodilatory effects in the pulmonary vascular system of Sacubitril/Valsartan may both in the end contribute to positive hemodynamic effects also in HFpEF patients. It should be noted that all patients in our case series were on a stable therapy with uptitrated dosages of angiotensin-converting enzyme inhibitors or angiotensin receptor blockers beforehand, while the second RHC was performed with a median Sacubitril/Valsartan dosage of only 24/26 mg bid. Further trials will thus have to clarify, whether higher dosages of Sacubitril/Valsartan will even display stronger effects on pulmonary pressures in HFpEF patients.

It should, however, be acknowledged, that this is a small single-center retrospective observational study and thus only generates a working hypothesis.

## Conclusions

Our data insinuate that Sacubitril/Valsartan might prove to be a novel therapeutic approach in HFpEF patients to treat pulmonary hypertension. Larger trials employing invasive phenotyping of pulmonary hemodynamics are thus eagerly awaited to solve an ever growing problem imposing a heavy burden of mortality and morbidity.

## Data Availability Statement

The raw data supporting the conclusions of this article will be made available by the authors, without undue reservation.

## Ethics Statement

The study protocol was approved by an institutional review and safety board at the Heart and Vascular Center Bad Bevensen. Written informed consent was not required for this study, in accordance with the local legislation and institutional requirements.

## Author Contributions

Data acquisition, analysis, and interpretation was done by JB, HS, and MJ. CB and BR drafted the manuscript. JB, HS, and MJ revised the article for important intellectual content. All authors gave final approval of the version to be published, involved in the conception of the present study, and fully accountable for the content of the manuscript.

## Conflict of Interest

The authors declare that the research was conducted in the absence of any commercial or financial relationships that could be construed as a potential conflict of interest.

## Publisher's Note

All claims expressed in this article are solely those of the authors and do not necessarily represent those of their affiliated organizations, or those of the publisher, the editors and the reviewers. Any product that may be evaluated in this article, or claim that may be made by its manufacturer, is not guaranteed or endorsed by the publisher.
